# Right diaphragmatic injury and lacerated liver during a penetrating abdominal trauma: case report and brief literature review

**DOI:** 10.1186/1749-7922-9-33

**Published:** 2014-04-28

**Authors:** Antonino Agrusa, Giorgio Romano, Daniela Chianetta, Giovanni De Vita, Giuseppe Frazzetta, Giuseppe Di Buono, Vincenzo Sorce, Gaspare Gulotta

**Affiliations:** 1 Department of General Surgery, Urgency and Organ Transplantation, University of Palermo, Via L. Giuffrè, Palermo 5 90127, Italy

**Keywords:** Diaphragmatic injury, Penetrating abdominal trauma, Diaphragmatic repair, Liver laceration, Stab wound

## Abstract

**Introduction:**

Diaphragmatic injuries are rare consequences of thoracoabdominal trauma and they often occur in association with multiorgan injuries. The diaphragm is a difficult anatomical structure to study with common imaging instruments due to its physiological movement. Thus, diaphragmatic injuries can often be misunderstood and diagnosed only during surgical procedures. Diagnostic delay results in a high rate of mortality.

**Methods:**

We report the management of a clinical case of a 45-old man who came to our observation with a stab wound in the right upper abdomen. The type or length of the knife used as it was extracted from the victim after the fight. CT imaging demonstrated a right hemothorax without pulmonary lesions and parenchymal laceration of the liver with active bleeding. It is observed hemoperitoneum and subdiaphragmatic air in the abdomen, as a bowel perforation. A complete blood count check revealed a decrease in hemoglobin (7 mg/dl), and therefore it was decided to perform surgery in midline laparotomy.

**Conclusion:**

In countries with a low incidence of inter-personal violence, stab wound diaphragmatic injury is particularly rare, in particular involving the right hemidiaphragm. Diaphragmatic injury may be underestimated due to the presence of concomitant lesions of other organs, to a state of shock and respiratory failure, and to the difficulty of identifying diaphragmatic injuries in the absence of high sensitivity and specific diagnostic instruments. Diagnostic delay causes high mortality with these traumas with insidious symptoms. A diaphragmatic injury should be suspected in the presence of a clinical picture which includes hemothorax, hemoperitoneum, anemia and the presence of subdiaphragmatic air in the abdomen.

## Background

Diaphragmatic injuries are a diagnostic and therapeutic challenge for the surgeon. They are often un recognized, and diagnostic delay causes high mortality from these injuries [1]. In countries with a low incidence of inter-personal violence, it is quite a rare trauma, with only 4-5% of patients undergoing laparotomy for trauma presenting a diaphragmatic injury [2]. These are mainly caused by blunt trauma of the chest and abdomen (75%) and, more rarely, by penetrating ones (25%) [3]. Clinical presentation varies from a state of hemodynamic instability secondary to bleeding of the diaphragm and organs involved in the trauma [4] to a condition of intestinal obstruction and respiratory failure that can occur months, or even years, after the trauma, due to diaphragmatic hernia [5]. Diagnosis is made difficult both by the frequent presence of concomitant multi-organ injuries that deviate the surgeon’s attention from the diaphragm, and by the lack of adequate diagnostic imaging studies regarding the diaphragmatic muscle. In hemodynamically stable patients with penetrating wound of the abdomen, in which there is a strong suspicion of diaphragmatic injury, with a given negative diagnostic imaging, laparoscopy is considered a valuable diagnostic and therapeutic tool in the presence of experienced surgeons. In hemodynamically unstable patients a midline laparotomy is the recommended approach as it allows exploration of the entire abdominal cavity [6].

## Methods

We report the clinical case of a 45 year-old man who came to our observation with a stab wound in the right upper abdomen, without cyanosis or dyspnea. Blood pressure was 130/80 mmHg and hemoglobin 12.5 mg/dl. On clinical examination, the patient had a lacerated, bleeding stab wound in the right upper quadrant through which part of the omentum, without other macroscopically visible injuries, could be seen. The type or length of the knife used as it was extracted from the victim after the fight. A focused assessment with sonography for trauma (FAST) test was carried out which showed subdiaphragmatic and perihepatic blood. Due to abundant tympanites and lack of cooperation on the part of the patient, nothing more could be seen. It was decided to have to patient undergo a CT scan of the abdomen to determine if there were any lesions to the abdominal organs.

**Figure 1:**
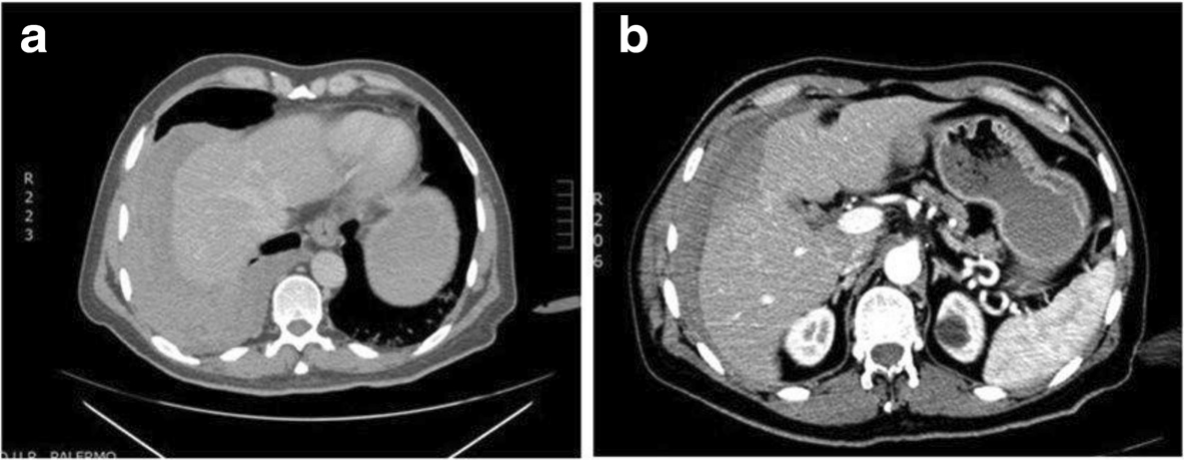
**Computed tomography results of the patient. a)** presence of a right hemothorax without pulmonary lesions; **b)** discrete hemoperitoneum by an active bleeding parenchymal liver laceration and “free air” in the abdomen.

**Figure 2:**
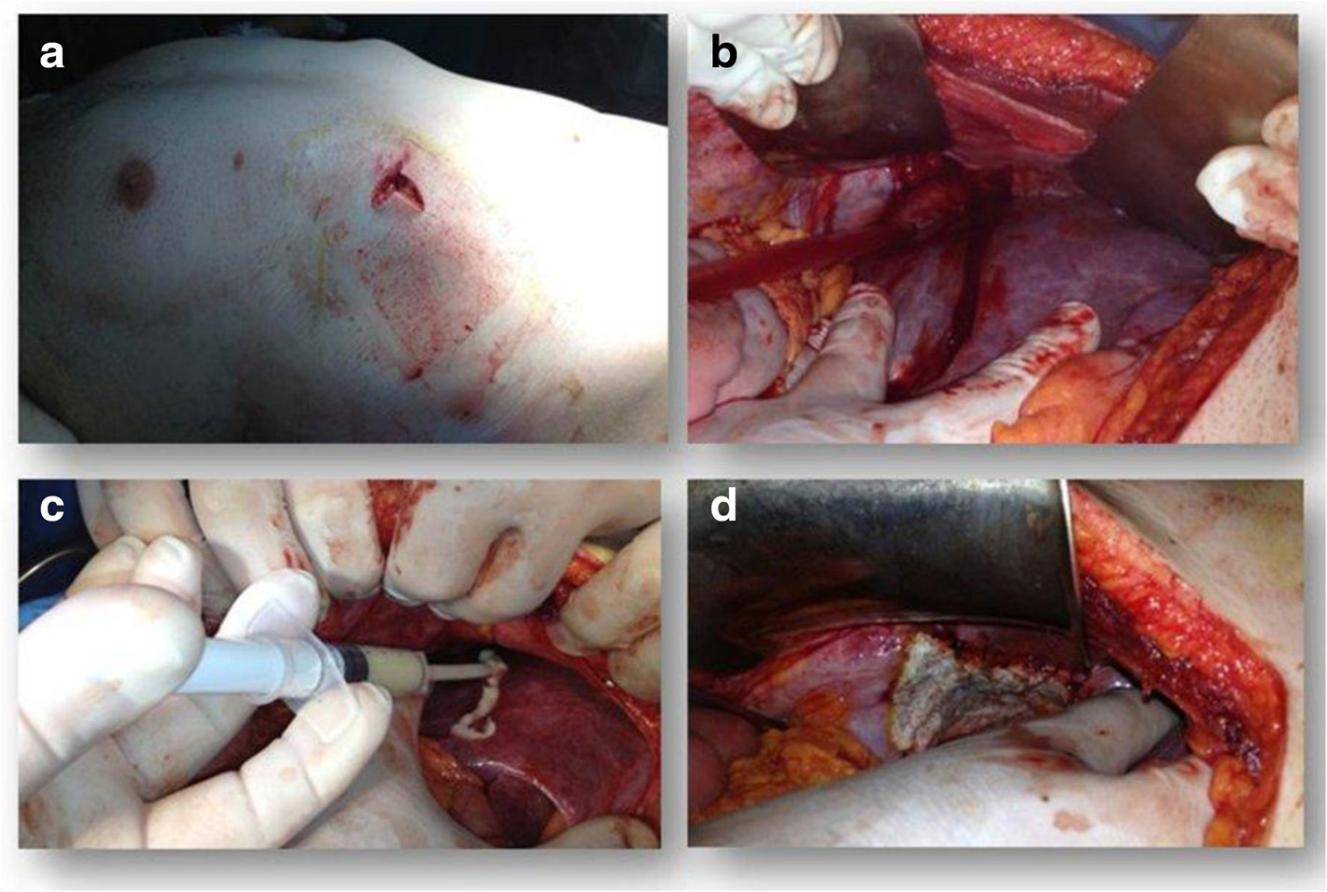
**Characteristics of the stab wound and intra-operative findings. a)** bleeding stab wound in the right upper quadrant; **b)** liver laceration and right diaphragmatic injury; **c)** application of hemostatic matrix (Floseal®) on liver lesion; **d)** repair of diaphragmatic lesion with non-absorbables sutures and positioning of medicated sponge containing thrombin and human fibrinogen (Tachosil®).

From the scan, the presence of a right hemothorax without pulmonary lesions was seen, with moderate hemoperitoneum from an active bleeding parenchymal liver laceration and subdiaphragmatic air in the abdomen as a bowel perforation (Figure 1). Initially, the suspect of a bowel perforation suggested a laparoscopic approach, but the patient’s hemodynamic condition rapidly changed. In the operating room, the patient presented pale with tachycardia; blood pressure decreased to 90/60 mm Hg and cardiac frequency increased to 115 bpm. A complete blood count check revealed a decrease in hemoglobin (7 mg/dl), and therefore it was decided to perform surgery in midline laparotomy [6,7]. After laparotomy, a significant amount of blood was evacuated to identify the site of bleeding. Liver inspection showed an 8 cm long, 1 cm deep laceration with active bleeding in segments IV-V (Grade II lesion classification AAST). A careful inspection of the abdominal cavity also showed a 12 cm length right diaphragmatic lesion with signs of active bleeding that accounted for the presence of free air seen in the CT images. No other intestinal lesions were found. Temporary packing was used to treat the liver bleeding. After evacuating the right hemothorax, we proceeded with repair of the diaphragmatic lesion with non-absorbable sutures, and by placing a thoracic Bouleau drainage. The suture was completed applying a medicated sponge containing thrombin and human fibrinogen in order to control hemostasis and facilitate the building of the tissues and healing process [8]. After stopping the bleeding from the liver and bile leakage it was decided to adopt a conservative approach applying hemostatic matrix on liver injury (Figure 2). Surgery was concluded with the placement of abdominal drains, in the right subphrenic space. One transfusion was carried out during surgery. In post-operative time, blood pressure was 120/80 mmHg, hemoglobin 9 mg/dl. Chest tube was removed 4 days post surgery, after an x-ray which confirmed resolution of hemopneumothorax.

## Discussion

The diaphragm is the principle muscle of respiration. With the contraction of striated muscle fibers it carries more than 70% of the work creating a negative intrathoracic pressure which is necessary for the proper performance of respiratory mechanics, as well as encouraging proper venous return to the heart. The integrity of the diaphragm separates the chest cavity from abdominal positive pressure, which ensures proper maintenance of the different pressure regimes of the two chambers, and prevents the migration of the abdominal organs into the chest. A laceration of the diaphragm produces an alteration of these physiological mechanisms with possible migration of the abdominal viscera into the thorax and the disappearance of the thoraco-abdominal pressure gradient which causes alteration of respiratory mechanics, compression of the vena cava with reduced venous return to the heart, and consequential respiratory failure and cardiovascular collapse [9]. Diaphragmatic rupture is a potentially lethal clinical condition for the patient and a delayed or missed diagnosis causes high mortality with this type of trauma [1].

In literature, the first description of diaphragmatic trauma dates back to the sixteenth century when in 1853 Bowditch described a diaphragmatic injury, in a dead victim of a gunshot penetrating trauma, during the autopsy [5]. The first repair with favorable outcomes of a penetrating diaphragmatic injury was described by Riolfi in 1886, while in 1900 Walker published the first repair of traumatic diaphragmatic gunshot lesion with favorable outcomes [10].

It is difficult to accurately estimate the real incidence of diaphragmatic injuries due to delayed or missed diagnosis and pre-hospital deaths [1]. Approximately 5% of patients with abdominal trauma at the time of thoracotomy or laparotomy have a diaphragmatic injury [2]. They are mainly caused by blunt trauma of the chest and abdomen (75%) and more rarely by stabbing (25%) [3]. Diaphragmatic injuries mainly affect the male sex (M/F ratio 3:1) generally occur following closed thoracoabdominal trauma and more rarely penetrating trauma [11]. Mortality rate ranges from 1% to 28%; this high percentage depends upon frequency of associated injuries but also on the delay between diagnosis and the traumatic event [3]. Diaphragmatic injuries frequently occur during automobile accidents; frontal impact causes an increase of intra-abdominal pressure resulting in a lesion in the radial wall posterolateral to the diaphragm [3]. Side impacts also may be associated with lesions of the liver or spleen in 96% of cases [11]. Diaphragmatic injuries during penetrating trauma of the abdomen are extremely rare, making up 25%, of which 20% from gunshot and 5% from weapon [3]. In the course of penetrating trauma to the abdomen small sized diaphragmatic lesions are often created, which may initially remain undetected and determinate the onset of a diaphragmatic hernia. Right hemidiaphragm trauma is less frequent than left trauma (with a ratio of 1:3) and also is diagnosed with greater delay. This is due to the protective function of the liver which lies on the right abdominal surface preventing herniation of the abdominal viscera into the thorax [9]. Furthermore, many studies performed on cadavers show that during closed trauma the pressure required to determine a lesion of the left hemidiaphragm is less than that required for the right side. [12]. Any discontinuity of the diaphragm leads to alterations of mechanical respiration and circulatory collapse until cardio circulatory system [13]. In severe multiple trauma with patient in a state of shock, respiratory failure and/or coma, diaphragmatic injuries can be misunderstood, as often the attention of the medical team is on damage to other organs which often occur in the course of this type of trauma. In acute phases, diaphragmatic rupture usually occurs with thoraco-abdominal pain, hypotension, hemodynamic instability, dyspnea, and cyanosis. Hemodynamic instability and shock are often the result of associated injuries and bleeding of the diaphragmatic muscle injury [14]. When the diaphragmatic lesion is small, it may go unrecognized for several hours, weeks or even months and manifest late and progressively as a diaphragmatic hernia with the appearance of typical symptoms of intestinal obstruction, tachycardia, dyspnea [15]. Small injury of the right hemidiaphragm may even remain undetected due to the protective function offered by the liver, which prevents bowel herniation into the thorax cavity. There is rarely herniation of the liver [16]. Preoperative diagnosis of diaphragmatic injury still represents a diagnostic challenge for the radiologist. The high mortality of this trauma is also linked to the difficulty of studying this anatomical site in emergency conditions [1]. In a chest x-ray, a diaphragmatic injury should be suspected when the hemidiaphragm is not correctly placed. The specific signs of a diaphragmatic lesion on chest x-rays are represented by the presence of air-fluid levels in the chest and the salience of a hemidiaphragm compared to the contralateral side. Chest x-ray has a diagnostic accuracy of less than 40% and can only detect indirect signs described, the absence of which does not rule out a diaphragmatic lesion [17]. Diagnostic accuracy is four times greater for lesions of the left hemidiaphragm (42%) compared to the right (17%) [8]. Chest x-ray has been replaced by computed tomography (CT) which has a diagnostic sensitivity of 50% for right hemidiaphragm lesions and of 70% for the left side ones. It allows the physician to see any discontinuity of the diaphragmatic profile and the presence of loops or omentum in the thoracic cavity, as well as the presence of hemoperitoneum and hemothorax [17]. Historically, CT showed poor visualization of the diaphragm due to motion of the muscle itself, but the advent of multiphasic spiral CT has led to a sensitivity of 80% and a specificity of 90% [18]. CT is a valuable diagnostic tool, readily available in trauma centers and executable in hemodynamically stable patients with multiple trauma. In hemodynamically unstable patients, ultrasound (US), and in particular FAST in real time can demonstrate the absence or reduced motility of the diaphragm suggestive of lesions of the muscle itself, with an accuracy of 30%. In addition, the US can identify the presence of indirect signs such as hemothorax and hemoperitoneum [19]. Magnetic resonance imaging (MRI) has a sensitivity and specificity of 95% in identifying a diaphragmatic lesion, it is not always available in emergency rooms, but extremely helpful in the diagnosis of post-traumatic diaphragmatic hernias [20].

Thus, in the absence of a bowel herniation through the lesion, it is very difficult to diagnose a diaphragmatic lesion with the conventional images that are readily available in emergency conditions [21]. This observation is even more valid when penetrating injuries affect the right upper quadrant of the abdomen. In these cases, the liver, due to its particular anatomical position, stands between the lesion and the viscera preventing diaphragmatic herniation of the latter into the chest through the opening in the diaphragm, accounting for the delay in diagnosis of this type of diaphragmatic injury [22]. In this case, there are indirect signs such as effusion into the thorax and abdomen, principally if there is a lacerated liver (98% of cases) and the presence of subdiaphragmatic air in the abdomen. In hemodynamically stable patients with penetrating injury of the abdomen in which there is a strong clinical suspicion of diaphragmatic hernia, laparoscopy is indicated as, in addition to having a diagnostic role [6,23] inidentifying the presence of associated lesions, when possible, it also allows repair of the torn diaphragm with a non-absorbable suture sutures [6]. In hemodynamically unstable patients a midline laparotomy is the recommended approach as it allows exploration of the entire abdominal cavity. The diaphragmatic lesion is repaired with non-absorbable suture after placement of chest tube.

In countries with a low incidence of inter-personal violence, stab wound diaphragmatic injury is particularly rare, in particular involving the right hemidiaphragm. Diaphragmatic injury may be underestimated due to the presence of concomitant lesions of other organs, to a state of shock and respiratory failure, and to the difficulty of identifying diaphragmatic injuries in the absence of high sensitivity and specific diagnostic instruments. Diagnostic delay causes high mortality with these traumas with insidious symptoms. A diaphragmatic injury should be suspected in the presence of a clinical picture which includes hemothorax, hemoperitoneum, anemia and the presence of subdiaphragmatic air in the abdomen.

## Consent

Written informed consent was obtained from the patient for publication of this case report and accompanying images. A copy of the written consent is available for review by the Editor-in-Chief of this journal on request.

## Competing interests

The authors declare that they have no competing interests.

## Authors’ contributions

AA, RG and CD study design and writing; DVG, FG, DBG and SV data analysis and writing; GG study the design. All authors read and approved the final manuscript.

## Authors’ information

Agrusa Antonino and other co-authors have no study sponsor.
